# Ultrasonic detection of fetal persistent right umbilical vein and incidence and significance of concomitant anomalies

**DOI:** 10.1186/s12884-020-03310-2

**Published:** 2020-10-09

**Authors:** Jingyu Li, Qian Yuan, Hao Ding, Zeyu Yang, Bing Wang, Bin Wang

**Affiliations:** 1 Department of Ultrasound, Shengjing Hospital of China Medical University, Shenyang, Liaoning China; 2 Department of Orthopaedics, Shengjing Hospital of China Medical University, 36 Sanhao St, Shenyang, 110004 Liaoning China

**Keywords:** Persistent right umbilical vein, Concomitant anomalies, Ultrasound, Prenatal diagnosis, Prognosis

## Abstract

**Background:**

Persistent right umbilical vein (PRUV) is characterized by atresia of the left umbilical vein while the right umbilical vein remains open. Given the limited sample size of most studies, the incidence of PRUV and the status of concomitant anomalies may not be fully reflected. Thus, we studied the incidence of fetal PRUV and its concomitant anomalies on a larger scale using our hospital database. This study hoped to address the following questions: Does PRUV increase the risk of fetal anomalies? If the PRUV fetus also has a single umbilical artery (SUA), does the risk of fetal anomaly increase further? What is the positive predictive value of PRUV for fetal anomalies?

**Methods:**

This retrospective study analyzed 756 cases of fetal PRUV at our hospital from January 2007 to April 2017. Prenatal ultrasound and color Doppler images were assessed. All PRUV fetuses underwent echocardiography and detailed ultrasound examinations of other systems. Newborn status was obtained via the database or by telephone follow-up.

**Results:**

A total of 435,428 pregnant women underwent prenatal ultrasonography at 16–40 weeks, the incidence of fetal PRUV was 0.17%, and 102 fetuses (13.5%) developed other anomalies. Two complicated cases had trisomy 18. PRUV was associated with a higher incidence of fetal anomalies. When fetal anomalies were classified by body systems, PRUV was associated with a higher incidence of cardiovascular, nervous, urinary, skeletal, digestive, and respiratory system anomalies. The positive predictive values of a PRUV for any fetal anomalies and cardiovascular anomalies were 13.5% (95%CI, 11.2–16.2%) and 5.4% (95%CI, 4.0–7.3%), respectively. SUA further increases the risk of PRUV fetuses with other anomalies and cardiovascular anomalies.

**Conclusions:**

Detailed prenatal ultrasonography and echocardiography should be performed in fetuses with PRUV to rule out anomalies in other systems. When the PRUV is combined with SUA, echocardiography is particularly important. Fetuses with complicated PRUV should undergo chromosomal examination. Although isolated fetal PRUV prognosis is good, complicated PRUV prognosis depends on the type and severity of the concomitant anomalies.

## Background

Persistent right umbilical vein (PRUV) is an embryonic vascular developmental abnormality in which there is atresia of the left umbilical vein, and the right umbilical vein remains open [1]. In early embryonic development, the umbilical vein has left and right branches that develop from the chorion, originate from the placenta, pass through the umbilical cord into the body of the embryo, and enter the sinus venosus through the primordial septum transversum. Under normal conditions, the right umbilical vein gradually begins to undergo atresia at embryonic week 4 and disappears completely by week 7. The segment of the left umbilical vein proximal to the heart, that is, the left umbilical vein between the liver and the sinus venosus, also degenerates, and the left umbilical vein from the umbilicus to the liver remains, which communicates with the umbilical vein in the umbilical cord, returning blood from the placenta to the inferior vena cava through the ductus venosus formed through the liver [2–5]. As a result, PRUV occurs if the left umbilical vein atrophies and degenerates and the right umbilical vein is retained [6].

PRUV can be diagnosed during prenatal ultrasonography by examination of the transverse section of the fetal abdomen. In 1990, Jeanty first reported the diagnosis of PRUV by prenatal ultrasonography [7]. At that time, PRUV was considered a rare abnormality and was often combined with malformations in other systems. In recent years, with improved awareness of fetal venous system examination and the advancement of ultrasound technology, studies found that PRUV is not a very rare condition, with researchers reporting an incidence of 0.08–0.5% [1, 2, 6, 8–12]. In addition, most cases of PRUV do not include malformations in other systems, with a concomitant malformation rate of 13–40.9% [1, 2, 6, 9, 10, 12]. Common malformations include cardiac, urinary, nervous, skeletal, and other malformations.

Thus far, the largest sample size reported for prenatal ultrasound diagnosis of PRUV is 313 cases [8]. Given the limited sample size of most studies, the incidence of PRUV and the status of concomitant anomalies may not be fully reflected. Thus, this study aimed to investigate the incidence of fetal PRUV by prenatal ultrasound examination using a larger sample size and to analyze which other anomalies were found in prenatal ultrasound examination of fetal PRUV. We also hope to address the following questions: Does PRUV increase the risk of fetal anomalies? If the PRUV fetus also has a single umbilical artery (SUA), does the risk of fetal anomaly increase further? What is the positive predictive value of PRUV for fetal cardiovascular anomalies?

## Methods

We retrospectively analyzed 756 cases of fetal PRUV at our hospital from January 2007 to April 2017 with records in the Picture Archiving and Communication System (PACS) database of the Department of Ultrasound. During the study period, 435,428 women with high-risk and low-risk pregnancies underwent prenatal ultrasonography at 16–40 weeks of gestational age. Fetuses with situs inversus, situs indeterminatus, and heterotaxy were excluded, because the left and right of the fetus in these cases are difficult to define.

From January 2016 to May 2016, 15,411 low-risk pregnant women with ultrasound data at 16–40 weeks in our hospital were selected as the control group.

**Fig. 1:**
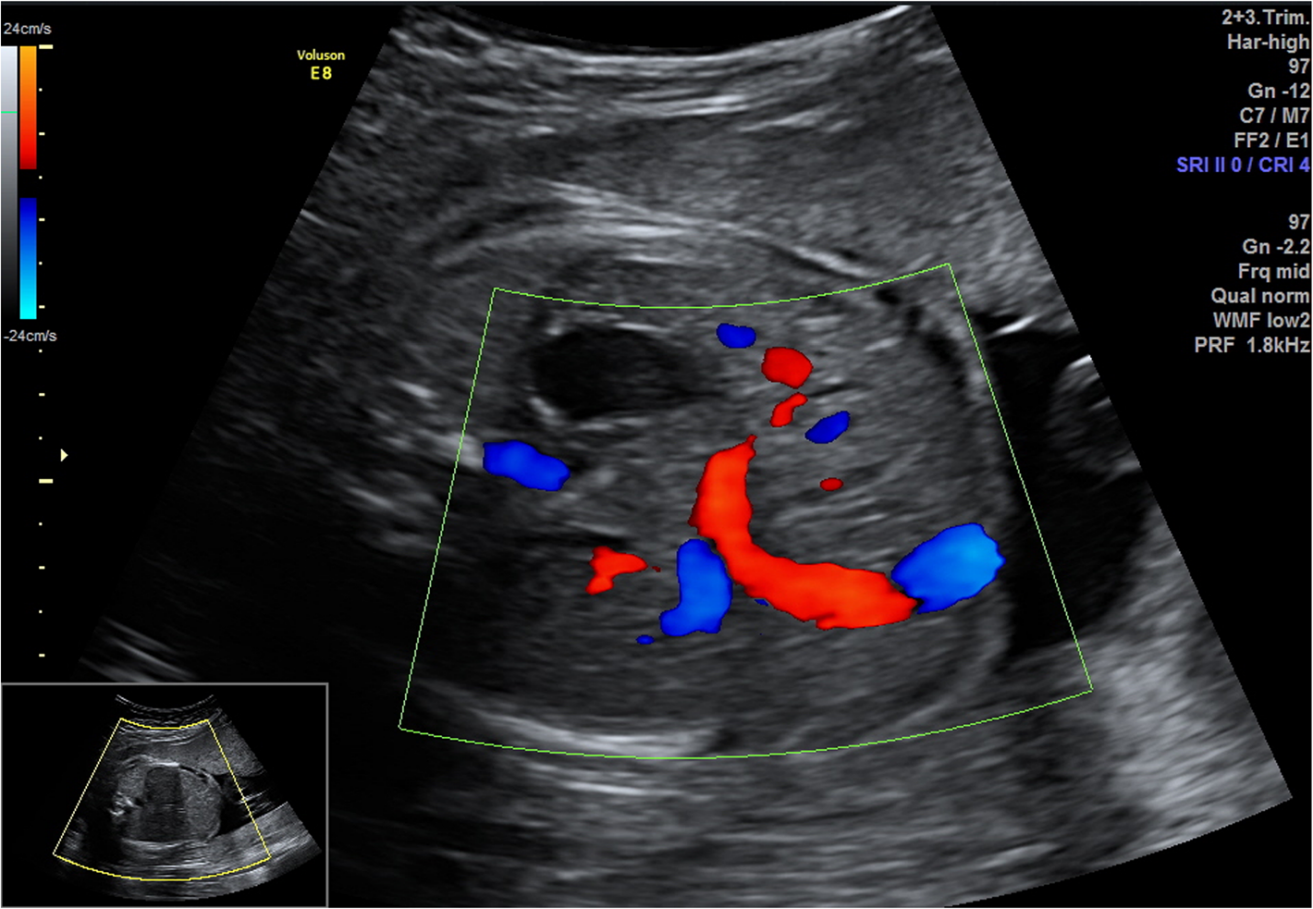
The umbilical vein bending toward the fetal stomach in the fetal abdominal circumference section

**Fig. 2:**
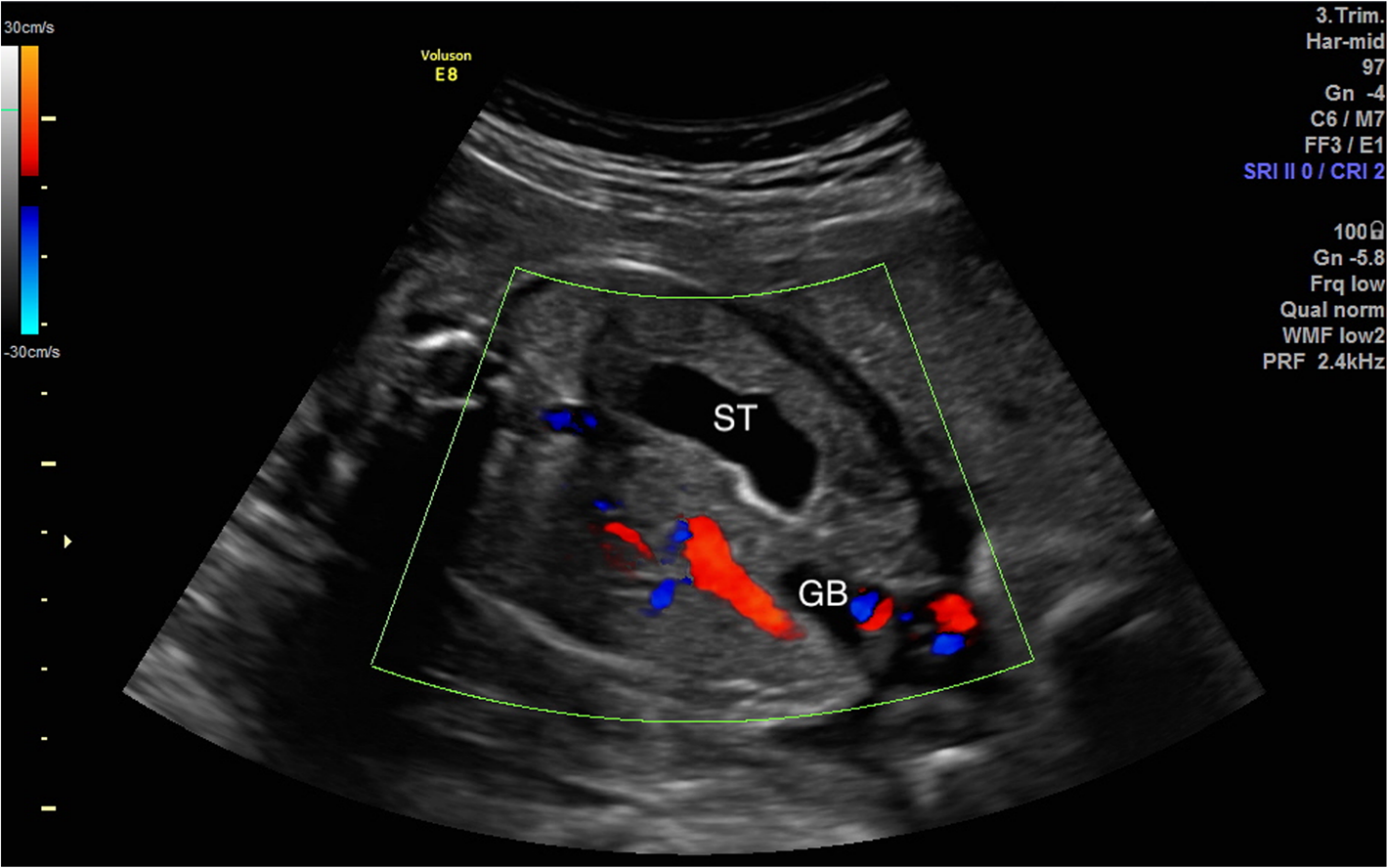
The gallbladder located between the umbilical vein and the stomach. GB, gallbladder; ST, stomach

Routine examinations and measurements of the fetus and adnexa were performed, and standard sections were stored in the PACS. The standard section at abdominal circumference was used to show the fetal stomach and the umbilical vein. The relationship between the route of the umbilical vein and the stomach and the gallbladder was observed. Color and spectral Doppler imaging were performed to detect the umbilical vein in the transverse and longitudinal sections. The ultrasound diagnostic criteria for fetal PRUV included the umbilical vein bending toward the fetal stomach in the fetal abdominal circumference section (Fig. 1), and the gallbladder located between the umbilical vein and the stomach (Fig. 2). All fetuses diagnosed with PRUV underwent fetal echocardiography and detailed ultrasound examinations of other systems to determine whether other concomitant anomalies were present.

The instruments used were the Voluson 730, Voluson E8, and Voluson E10 (GE Healthcare Technologies, Milwaukee, WI, USA), all equipped with convex array two- and three-dimensional abdominal transducers. All diagnoses of PRUV and concomitant anomalies were reviewed by two individuals with prenatal diagnostic qualifications. The study was reviewed by the Hospital Human Subjects Ethics Committee, Shengjing Hospital of China Medical University.

Detailed maternal and fetal hospitalization records for the perinatal period of pregnant women who gave birth at our hospital were available in the PACS. Pregnant women who did not give birth in our hospital were also followed up by telephone to ascertain the condition of the fetus with PRUV at birth. Data obtained through telephone follow-up were as follows: gestational age at birth, mode of delivery, neonatal birthweight, admission to the neonatal intensive care unit, and developmental anomalies and malformations confirmed by medical examination. In the latter two cases, patient family members were asked to provide detailed hospitalization records, discharge summary documents, and related medical examination reports.

Data were expressed as mean ± SD for normal distributions. The Statistical Package for Social Sciences version 19.0 (SPSS Inc., Chicago, IL, USA) was used for statistical analysis.

## Results

A total of 756 cases of PRUV were identified from 435,428 pregnancies, obtaining an incidence rate of 0.17%. Most of the cases (*n* = 654, 86.5%) were of isolated PRUV, in which no additional anomalies (excluding isolated SUA) were found on prenatal ultrasound examination. However, 102 cases were complicated PRUV (13.5%) in which additional anomalies were found on prenatal ultrasound examination. The demographic characteristics of these fetuses with PRUV are summarized in Table 1. The pregnant women were 17–45 years old, and gestational age at diagnosis ranged from 16.0 to 40.4 weeks.

**Table 1:** Demographic characteristics of patients with PRUV

Characteristic	Total	Isolated	Complicated
Age, years	28.8 ± 4.5	28.8 ± 4.4	28.7 ± 4.6
Gestational age at examination, weeks	28.0 ± 4.8	28.0 ± 4.6	27.8 ± 5.5
BMI	25.8 ± 3.9	25.8 ± 3.8	25.9 ± 4.2
Singleton, n	719	622	97
Twin, n	37	32	5
Primipara, n	615	533	82
Multipara, n	141	121	20
Birth weight, g	3023 ± 482	3134 ± 453	2878 ± 590

Concomitant anomalies in 102 fetuses with complicated PRUV are listed in Table 2. These included 41 cardiovascular (40.2%), 22 nervous (21.6%), 17 urinary (16.7%), 15 skeletal (14.7%), five digestive (4.9%), and four respiratory anomalies (3.9%) and 29 other anomalies (some fetuses had multiple concomitant anomalies). The positive predictive values of a PRUV for any fetal anomalies and cardiovascular anomalies were 13.5% (95% confidence interval [CI], 11.2–16.2%) and 5.4% (95%CI, 4.0–7.3%), respectively.

**Table 2:** Concomitant anomalies in complicated PRUV cases

Body System	Type of malformation	Number of cases
Cardiovascular		41
	Ventricular septal defect	10
	Persistent left superior vena cava	9
	Single ventricle	7
	Complete atrioventricular canal defect	6
	Common arterial trunk	6
	Transposition of the great arteries	6
	Right-sided aortic arch	4
	Tetralogy of Fallot	4
	Pulmonary artery stenosis	3
	Aberrant left subclavian artery	2
	Dextroversion of heart	2
	Double outlet right ventricle	1
	Right heart enlargement, tricuspid valve regurgitation	1
	Dextrocardia	1
	Interrupted aortic arch	1
	Hypoplastic left heart	1
	Atrial septal defect	1
	Pulmonary artery valve stenosis	1
	Total anomalous pulmonary venous return	1
Nervous system		20
	Spina bifida	5
	Choroid plexus cyst	5
	Hydrocephalus	3
	Cerebellar vermis agenesis, incomplete development	2
	Vein of Galen malformation	2
	Posterior fossa effusion	1
	Lateral ventricular dilatation	1
	Bilateral ependymal cyst	1
	Bilateral lateral intraventricular hemorrhage	1
	Agenesis of the corpus callosum	1
	Anencephaly	1
Urinary system		17
	Unilateral or bilateral hydronephrosis	5
	Unilateral duplex kidney or double pelvis	3
	Unilateral or bilateral renal agenesis	3
	Unilateral multicystic dysplastic kidney	3
	Right renal hypoplasia	1
	Ectopic kidney in right pelvic cavity	1
	Right renal cyst	1
	Ureterocele	1
	Bilateral renal echo enhancement	1
Skeletal system		17
	Shortened limbs	8
	Unilateral or bilateral club hand	5
	Bilateral clubfoot	2
	Unilateral shortened ulna and radius	2
	Agenesis of right upper arm and forearm (right hand directly connected to shoulder joint)	1
	Hemivertebrae	1
	Butterfly vertebrae	1
Digestive system		5
	Stenosis or atresia of small bowel	2
	Esophageal atresia	2
	Intestinal duplication	1
Respiratory system		4
	Pulmonary cystic adenomatoid malformation	1
	Pulmonary sequestration	1
	Left lung agenesis	1
	Left lung dysplasia	1
Other anomalies		29
	Diaphragmatic hernia	5
	Cleft lip and palate	3
	Choledochal cyst	3
	Ovarian cyst	3
	Intestinal echo enhancement	3
	Fetal intraabdominal umbilical vein varix	2
	IUGR	2
	Umbilical cord cyst	2
	Intrauterine fetal death	1
	Mandibular hypoplasia	1
	Cystic teratoma in abdominal cavity	1
	Orbital hypertelorism	1
	Short umbilical cord	1
	Acromphalus	1
	Hepatic cyst	1
	Multiple echo enhancement foci in gallbladder	1
	Intrahepatic bile duct dilatation	1

Some cases had multiple concomitant malformations

PRUV was associated with a higher incidence of any fetal anomalies (odds ratio [OR], 5.42; 95% CI, 4.32–6.82). When fetal anomalies were classified by systems, PRUV was associated with a higher incidence of anomalies in the cardiovascular system (OR, 9.15; 95%CI, 6.3–13.29), nervous system (OR, 6.76; 95%CI, 4.16–11.0), urinary system (OR, 7.7; 95%CI, 4.39–13.49), skeletal system (OR, 8.41; 95%CI, 4.6–15.4), digestive system (OR, 12.82; 95%CI, 4.18–39.28) and respiratory system (OR, 3.72; 95%CI, 1.28–10.82).

A total of 41 PRUV fetuses in this group had concomitant cardiac malformations. Among them, cardiac malformations were detected by obstetric ultrasound in 29 cases, which were consistent with results from fetal echocardiography. In 10 cases, cardiac malformations were detected with obstetric ultrasound; however, the malformation types could not be determined and were therefore confirmed using fetal echocardiography with the following results: 1case of single ventricle (SV), complete transposition of the great arteries (TGA) and total anomalous pulmonary venous; 1 case of interrupted aortic arch, hypoplastic left heart and persistent left superior vena cava (PLSVC); 1 case of atrial septal defect and common arterial trunk (CAT); 1 case of double outlet right ventricle, ventricular septal defect (VSD) and pulmonary artery stenosis (PAS); 1 case of right-sided aortic arch and aberrant left subclavian artery; 1 case of CAT and VSD; 1 case of SV and pulmonary artery valve stenosis; 1 case of complete atrioventricular canal defect (ACD) and PAS; 1 case of complete ACD and CAT; and 1 case of tetralogy of Fallot. Cardiac malformations could not be detected in 2 cases by obstetric ultrasound, but were later confirmed with fetal echocardiography, including 1 case of perimembranous VSD and 1 case of PLSVC.

In 756 PRUV fetuses, 31 developed SUA, of which 16 had other anomalies (10 had cardiovascular anomalies). In PRUV fetuses, SUA was associated with a higher incidence of any concomitant anomalies (OR, 7.93; 95%CI, 3.78–16.6) and a higher incidence of cardiovascular anomalies (OR, 10.66; 95%CI, 4.63–24.56). Moreover, the sensitivity and positive predictive values of SUA for any concomitant anomalies were 15.7% (95%CI, 9.5–24.5%) and 51.6% (95%CI, 33.4–69.4%) and those for cardiovascular anomalies were 24.3% (95%CI, 12.9–40.6%) and 32.3% (95%CI, 17.3–51.5%), respectively. Among the 31 PRUV fetuses with concomitant SUA, 9 underwent prenatal chromosomal examinations. Of those, 5 had concomitant SUA without other anomalies and chromosomal examination results that were normal. The remaining 4 fetuses had concomitant SUA and other malformations, including 2 with trisomy 18 and 2 that were normal (Table 3).

**Table 3:** Karyotypes of PRUV fetuses with concomitant single umbilical artery and other malformations

Case	Concomitant anomalies	Karyotype
1	bilateral choroid plexus cysts shortened right ulna and radius bilateral club hands intestinal echo enhancement	trisomy 18
2	posterior fossa effusion ventricular septal defect bilateral club hands local thickening of Wharton’s jelly in the umbilical cord	trisomy 18
3	agenesis of the corpus callosum	normal
4	ventricular septal defect ectopic kidney in right pelvic cavity	normal

A total of 189 PRUV fetuses underwent chromosomal examinations, including 2 aneuploid fetuses with multiple congenital anomalies (both were trisomy 18) (Table 3), and 187 PRUV fetuses with normal karyotypes (40 with complicated PRUV, and 147 with isolated PRUV). In addition, we found three cases of chromosome variation: 1 case of isolated PRUV with 46, XN, inv. (9) (p12q13), 1 case of complicated PRUV with 46, XN, inv. (9) (p11q13) presenting with spina bifida, and one case of complicated PRUV with 46, XN (15pslk+) (16qh+) presenting with cerebellar vermis agenesis, mandibular hypoplasia, acromphalus, bilateral club hands, a VSD, and TGA. (Chromosome tests in China do not report sex, so N represented chromosome X or Y.)

Of the 639 fetuses with isolated PRUV, 93 were lost to follow-up and 546 did not present any anomalies at birth. Of the 102 cases of complicated PRUV, 12 were lost to follow-up, including one intrauterine death with concomitant acromphalus, SUA, and umbilical cyst; 38 cases of termination of pregnancy (TOP); 1 neonatal death with diaphragmatic hernia and left lung dysplasia, eight postnatal surgeries, and 42 did not receive special treatment after birth. Among the 38 cases of TOP, we identified 2 trisomy 18 fetuses with multiple malformations (Table 3). The remaining 36 fetuses included 18 cardiac, 10 nervous, 8 skeletal, five urinary, and three digestive malformations. In addition, we found 8 cases of SUA, two cases of cleft lip and cleft palate, one case of acromphalus, one case of diaphragmatic hernia, and one case of left lung agenesis. (Some cases had multiple concomitant malformations.)

## Discussion

To the best of our knowledge, this study has the largest sample of prenatal ultrasound diagnoses of fetal PRUV to date, including 756 PRUV fetuses. This study analyzed and summarized 10 years of data from the prenatal ultrasound center of our hospital to find the incidence of PRUV (0.17%) and of other concomitant anomalies found on prenatal ultrasound examination of PRUV fetuses (13.5%). The most common types of anomalies in PRUV fetuses were cardiovascular anomalies, followed by nervous system, urinary system, skeletal and other anomalies. When a PRUV fetus has a SUA, the risk of any concomitant anomalies and the risk of fetal cardiovascular anomalies are further increased.

The prenatal ultrasound detection rate of fetal PRUV differs among studies. Several studies have shown detection rates similar to our rate of 0.17% [1, 6, 8, 12]. Retrospective studies in Spain and Germany had lower detection rates of 0.1 and 0.08% [2, 10]. Two studies showed much higher detection rates of 0.46 and 0.5%, but the authors have attributed this to the private system and referrals in one study and the prospective study design and small sample size in the other [9, 11]. The actual incidence may be slightly higher than 0.17% because our data includes late pregnancy and emergency cases. Given the limitations of fetal position and limited examination time, PRUV may be missed in such examinations. If the PRUV was associated with a significant anomaly, demise may have occurred before detection, thereby lowering the detection rate.

PRUV can appear in isolation or concomitantly with anomalies in other systems. Our study shows that approximately 13.5% of PRUV fetuses have anomalies in other systems, similar to the concomitant malformation rate of 13% reported by Blazer et al. [12] or 17.9% reported by Adiego-Calvo et al. [6], and was lower than the rate of 23.5–40.9% reported by other researchers [1, 2, 9, 10]. The lower proportion of PRUV fetuses with concomitant anomalies observed in our study may be due to the following: first, our study included cases at late gestation, while some fetuses with PRUV and concomitant serious anomalies might have been aborted during early or mid-gestation or had stopped growing in utero. Other studies focused on the second trimester window which may have detected more serious anomalies. Second, fetuses with isolated SUA were not included in the concomitant anomaly group in our study. By comparison, some studies included fetuses with isolated SUA in the concomitant anomaly group, thus increasing the proportion of fetuses with anomalies. Third, the discrepancy may be explained by different sample sizes.

Our study shows that compared with the control group, anomalies were increased in PRUV fetuses, and when these anomalies were classified by systems, the risk of cardiovascular nervous, urinary, skeletal, digestive, and respiratory system anomalies were increased. The most common anomalies in PRUV fetuses were cardiovascular system anomalies, followed by nervous system, urinary system, skeletal, and other anomalies. Similar to our study, Adiego-Calvo et al. [6] found a statistically significant association between isolated PRUV and the presence of congenital malformations, but in their series, only malformations of the genitourinary system showed this positive association. The difference in sample size may be the main reason for the different results.

When both PRUV and SUA occurred, the risk of any fetal anomalies and cardiovascular anomalies is further increased. Our study is the first to report this significant finding, which suggests that when PRUV and SUA coexist, detailed examination of other fetal systems, especially the cardiovascular system, should be conducted.

In this study, the presence of the most common anomalies suggest that PRUV may not only be a variation of the anatomy of the umbilical vein, but it may also be a link to developmental abnormalities of the fetal cardiovascular system, or it may be regulated by a common mechanism controlling the development of the nervous system, urinary system, and skeletal system; these speculations must be validated by in-depth studies. Other proposed causes in the literature include thromboembolism or external compression in early pregnancy leading to early occlusion of the left umbilical vein, preserving the blood supply to the right umbilical vein [1], insufficient folic acid supplementation, or effects of specific teratogenic drugs in early pregnancy [7].

In previous reports, chromosomal abnormalities in PRUV fetuses include trisomy 21, trisomy 18, Turner mosaicism, Noonan’s syndrome, and so on. Wolman et al. reported a case of a PRUV fetus with trisomy 18 [1], Bradley et al. reported a case of a PRUV fetus with Noonan’s syndrome [13], Weichert et al. reported a case of PRUV fetus with Turner mosaicism and another with trisomy 18 [10], Krzyżanowski et al. reported a case of a 69, XXX PRUV fetus [9], and Canavan et al. reported five cases of PRUV fetuses with chromosomal abnormalities (three cases of trisomy 18 and two cases of trisomy 21). Of these cases, only one case with trisomy 21 and one case with Turner mosaicism had no additional abnormal sonographic findings. In our cohort of patients, two cases of trisomy 18 were found, both of which had multiple malformations. Collectively, these reports and our results demonstrate that PRUV fetuses with chromosomal abnormalities usually have malformations of other systems, and isolated PRUV does not have a high risk of chromosomal abnormalities. PRUV fetuses with additional abnormal sonographic findings should undergo fetal chromosome examination.

This study has some limitations. First, this study has a retrospective design. Thus, some cases of fetal PRUV may not be detected due to the limitations of the fetal position during the third trimester and limited time during emergency ultrasound examination, which may result in an underestimated PRUV detection rate. Second, since we did not perform routine examinations of the fetal ductus venosus before October 2010, we were unable to classify the cases as intrahepatic or extrahepatic PRUV in this study. After October 2010, we confirmed the presence of the fetal ductus venosus with ultrasound examinations performed during the first and second trimesters. However, all the PRUV fetuses confirmed after October 2010 showed to be intrahepatic PRUV, and therefore it is likely that extrahepatic PRUV is extremely rare in our region. Based on the above reasons, it is certain that most of PRUV were intrahepatic in this study. Third, the abnormalities were found by ultrasonography, not the results of pathological examinations.

## Conclusions

In conclusion, fetuses with PRUV exhibit increased risk of concomitant anomalies. In addition, the coexistence of PRUV and SUA further increases the risk of other concomitant anomalies and cardiovascular anomalies in fetuses. When fetal PRUV is found on prenatal ultrasound examination, detailed examination of other fetal structures should be performed to rule out anomalies in other systems, and fetal echocardiography should be performed. Fetuses with complicated PRUV should undergo fetal chromosomal examination, whereas fetuses with isolated PRUV are not at high risk of chromosomal abnormalities. Isolated PRUV has a good prognosis, and the prognosis of complicated PRUV depends on the type and severity of the concomitant malformations. Whether PRUV is an anatomical variation or a developmental abnormality still warrants investigation in further studies.

## Data Availability

The datasets used and/or analysed during the current study are available from the corresponding author on reasonable request.

## Abbreviations

PRUV: Persistent right umbilical vein
SUA: Single umbilical artery
PACS: Picture Archiving and Communication System
CI: Confidence interval
OR: Odds ratio
SV: Single ventricle
TGA: Transposition of the great arteries
PLSVC: Persistent left superior vena cava
CAT: Common arterial trunk
VSD: Ventricular septal defect
PAS: Pulmonary artery stenosis
ACD: Atrioventricular canal defect
